# Pharmaceutical Preparations of Corn Silk

**Published:** 1883-08-20

**Authors:** George W. Kennedy


### PHARMACEUTICAL PREPARATIONS OF CORN SILK.

By George W. Kennedy, Ph.G.

Read at the Pharmaceutical Meeting, April 17.

During the past year several physicians of Schuylkill county have been using different preparations of the stigmata of Zea Mays for catarrh of the bladder and similar diseases with very good results. The preparations should be made from the fresh article, as the dried seems to be worthless; at least, that is the experience of those who have had the subject under investigation: cases under treatment, which were not benefitted by the powder or other preparations made from the dried article, yielded to a tincture prepared from the fresh or green stigmata. It would be advisable to gather the drug before it begins to change in color, or select only that portion having a green or greenish-yellow color. The writer manufactured a quantity of the tincture last September, which has all been prescribed and used by our physicians, and I am now compelled to purchase the fluid extract to supply the demands. One of our medical practitioners, who is very particular, has great confidence in the curative properties of corn silk; his choice of all the preparations is the syrup which I have made and would recommend to be made from the fluid extract. This is an expeditious mode of making the syrup, and one which is entirely satisfactory, the syrup containing only a very small percentage of alcohol. The diseases for which corn silk is recommended are of such a nature—generally of an inflammatory character—that the patient should not use alcohol in any form, because it produces irritation, and irritants should be left out of the preparations as much as possible.

Should the drug prove to be as valuable a remedy as some medical men consider it to be, there is no doubt but its use would become general. Either the fluid extract or the syrup, or both, would be the best preparations to recommend for introduction, although the tincture gave fair satisfaction, yet I do not believe it to be the most suitable preparation.

It should be remembered that the fresh drug contains a large amount of moisture; it contains certainly not less than fifty per cent., and likely considerably more. I would suggest that not less than double the quantity of the drug be used; for example, if a hundred parts of syrup or tincture was to represent twelve parts of the dried material, then twenty-four parts of the fresh or green corn silk should be used. I would recommend the following formulas:

### TINCTURE OF CORN SILK.

Take of corn silk, green, twenty-four parts.................. 24
" diluted alcohol sufficient to make one hundred parts. . 100

Cut the silk into small pieces, either with a large pair of scissors or a tobacco cutter; after which, place in a mortar and beat into a pulp with a small quantity of the diluted alcohol. Prepare a cylindrical glass percolator, by closing the lower orifice with a cork; transfer the silk pulp to the percolator, and add sufficient of the menstruum to form a layer over the pulp; cover the percolator closely and allow to macerate for forty-eight hours; then loosen the cork enough to permit percolation to proceed at the rate of forty drops per minute; add enough diluted alcohol and continue the percolation until one hundred parts are obtained. The tincture possesses the characteristic odor of corn silk, is of a yellow straw color and of a pleasant, sweetish taste. Dose for an adult, one or two fluid drachms (gm. 4—8).

### FLUID EXTRACT OF CORN SILK.

Corn silk, green, two hundred grammes....................200
Glycerin, twenty grammes.................................. 20
Diluted alcohol, a sufficient quantity to make one hundred centimeters..........................................100

Cut the silk into small pieces. Mix the glycerin with eighty grammes of diluted alcohol. Place the cut corn silk into a mortar, and beat into a pulp with a portion of the menstruum; after which, pack in a cylindrical glass percolator; add sufficient of the mixture to cover the pulpy mass, and when the liquid commences to drop from the percolator close the lower orifice; cover the percolator tightly, and allow to macerate for forty-eight hours, then permit percolation to go on slowly, about forty drops per minute; add the remainder of the glycerin mixture, and then diluted alcohol until the drug is exhausted, reserving the first seventy cubic centimeters of the percolate; evaporate the remainder to thirty cubic centimeters, and mix with the reserved portion, making in all one hundred cubic centimeters. The odor and taste is similar to that of the tincture, but much stronger, and a shade or two darker. Dose for an adult from half to one fluid drachm (gm. 2—4).

### SYRUP OF CORN SILK.

| | |
|---|---|
| Fluid extract of corn silk, twelve parts | 12 |
| Syrup, eighty-eight parts | 88 |
| To make one hundred parts | 100 |

Dose, from one to two fluid drachms.—*Jour. of Phar.*

